# A green approach for synthesizing zinc oxide nanoparticles utilizing aqueous extract of lemon peel: antibacterial evaluation and in silico analysis

**DOI:** 10.3389/fchem.2026.1880575

**Published:** 2026-09-03

**Authors:** Abdullah Mohammed Alrashadi, Arshid Nabi, Saleh Mohammed Al-Maaqar, Adeel Malik, Maqsood Ahmad Malik, Majid Rasool Kamli

**Affiliations:** 1 Department of Biological Sciences, Faculty of Science, King Abdulaziz University, Jeddah, Saudi Arabia; 2 Department of Chemistry, Sri Pratap College, Srinagar, Jammu and Kashmir, India; 3 Institute of Intelligence Informatics Technology, Sangmyung University, Seoul, Republic of Korea; 4 PAPRSB Institute of Health Sciences, Universiti Brunei Darussalam, Gadong, Brunei; 5 Department of Chemistry, Faculty of Sciences, Jamia Millia Islamia, New Delhi, India; 6 Center of Excellence in Bionanoscience Research, King Abdulaziz University, Jeddah, Saudi Arabia

**Keywords:** antibacterial agents, biosynthesis, phytochemicals, reducing agents, stabilizing agents, pectin, hesperidin, eriocitrin

## Abstract

**Introduction:**

Green-synthesized metal nanoparticles, primarily derived from plant sources, have attracted interest due to their inherent sustainability. In this study, we reported the green synthesis of zinc oxide nanoparticles (ZnO NPs) using an aqueous extract of dried lemon peel. In addition, antibacterial activity and *in silico* analysis of the as-prepared ZnO NPs were performed.

**Methods:**

Different techniques, including X-ray diffraction (XRD), field-emission scanning electron microscopy (FESEM), energy-dispersive X-ray spectroscopy (EDX), high-resolution transmission electron microscopy (HR-TEM), selected-area electron diffraction (SAED), Thermogravimetric Analysis (TGA) and Zeta potential were used to confirm the synthesis of ZnO NPs using lemon peel extract. MIC and disk diffusion assays were performed to evaluate the antibacterial activity of the ZnO NPs. In silico target prediction and protein–protein interaction (PPI) network analysis of phytochemical compounds present in the lemon peel extract were performed.

**Results:**

Multiple characterization techniques demonstrate the production of ZnO NPs from the lemon peel extract as a capping and stabilizing agent. The antibacterial efficacy of ZnO nanoparticles was assessed against six Gram-positive and Gram-negative bacterial strains, with minimum inhibitory concentrations (MICs) varying from 5 to 15 μg/mL. Furthermore, antimicrobial activity was evaluated by well and disc diffusion assays at different concentrations, confirming its antibacterial activity. Biofilm inhibition was observed at concentrations of 50 μg/mL and 75 μg/mL. In addition, *in silico* target prediction and PPI network analysis of major lemon peel-derived phytochemicals (eriocitrin, hesperidin, and pectin), which may remain associated with the biosynthesized nanoparticles as capping or stabilizing agents, revealed interactions with multiple bacterial proteins, particularly in *E*. *coli*, with significant enrichment in nucleotide biosynthesis, fatty acid metabolism, and central carbon metabolism pathways.

**Discussion:**

Overall, the results suggest that biogenic ZnO NPs exhibit strong antibacterial and antibiofilm activity. Computational analyses further indicate that major lemon peel-derived phytochemicals associated with nanoparticle synthesis may contribute to antibacterial activity through interactions with multiple bacterial metabolic pathways, alongside established ZnO-mediated mechanisms such as reactive oxygen species generation, membrane disruption, and zinc ion release.

## Introduction

Nanoparticles (NPs) are synthesized in ways distinct from conventional physico-chemical techniques. They have diverse applications across several domains, including life sciences, surface coatings, catalysts, food packaging, corrosion prevention, environmental remediation, electronics, biomedical applications, and antibacterial agents (Altammar, 2023; Vijayaram et al., 2024). Metallic nanoparticles and metal oxide nanostructures can be fabricated using physical and chemical methods, including sputtering, lithography, and electrospinning (Nyabadza et al., 2023). However, these methods are costly, and working with toxic chemicals can harm human health. Alternative biocompatible and green synthesis methods are adopted, with negligible use of chemicals, high-pressure reactors, or high temperatures, thereby enhancing yields. Most importantly, the green synthesis method produces less waste that is more readily biodegradable and less likely to pollute the environment (Kurul et al., 2025). Scientists have recently shown interest in green chemistry, synthesizing NPs containing naturally occurring biomass-derived active components from plants, fruits, flowers, algae, yeasts, bacteria, and fungi (Saxena et al., 2025). Additionally, extensive studies have been conducted using plant extracts to synthesizeNPs. It has been found that phyto-compounds are superior to other biomass-derived chemical components, even at the pilot scale, for the biosynthesis of NPs.

Eco-friendly technologies for preparing nanoscale materials have been a key focus of material scientists in recent years (Maind et al., 2025; Maurya et al., 2026). In the synthesis of NPs, green chemistry is gaining popularity for its reputation as inexpensive, safe, and easy to implement (Mechi et al., 2025; Ying et al., 2022). The use of agricultural waste in the synthesis of nanomaterials has emerged as a convergence of environmental sustainability and nanoscience (Abdelmonem et al., 2025; Barik et al., 2026; Wary et al., 2023). Due to its unique features and numerous applications, including medicine delivery, photocatalytic degradation, and personal care items such as sunscreens and cosmetics, zinc oxide (ZnO) has recently attracted the interest of many scientists for the biosynthesis of NPs (Anjum et al., 2021; Lu et al., 2015; Mousa et al., 2024; Deka et al., 2025). ZnO NPs have been biosynthesized using a variety of plant extracts, as reported in the literature (*Cassia auriculata* (Chaudhary et al., 2019; Prasad et al., 2020), *Pelargonium odoratissimum L.*) (Abdelbaky et al., 2022). ZnO is an ideal antibacterial material due to its wide bandgap, non-toxicity, and proven track record in applications (Sirelkhatim et al., 2015). Moreover, its unique antibacterial mechanisms stem from its nanoscale size. The bactericidal activity of ZnO nanoparticles (NPs) has been demonstrated against a wide range of bacteria, including Gram-positive and Gram-negative organisms such as *E. coli*, *S. aureus*, *P*. *aeruginosa*, and *K*. *pneumonia* (Siddiqi et al., 2018).

Zinc oxide (ZnO) nanoparticles are of significant interest among nanomaterials owing to their broad-spectrum antibacterial activity, superior biocompatibility, chemical stability, and very low toxicity (Raghupathi et al., 2011; Sirelkhatim et al., 2015). ZnO nanoparticles exhibit antimicrobial activity through multiple mechanisms, including the generation of reactive oxygen species (ROS), disruption of bacterial cell membranes, release of Zn^2+^ ions, and induction of oxidative stress, thereby complicating the development of bacterial resistance compared with traditional antibiotics (Jones et al., 2008).

Infectious diseases have posed significant challenges to human health for centuries and are regarded as the primary cause of human mortality. Antibiotics have significantly contributed to controlling infectious diseases and decreasing human mortality (Aminov, 2010; Hoffman, 2001). Antibiotics, whether bactericidal or bacteriostatic, have exerted evolutionary pressure on bacteria, leading to drug resistance in certain pathogens (Ahmed et al., 2024). On the other hand, new antibiotics are being developed slowly, while existing antibiotics are insufficient and are further limited by misuse, ultimately leading to antibiotic resistance (Malik and Bhattacharyya, 2019). Thus, developing novel antibiotics with alternative mechanisms of action can resolve these issues (Chavada et al., 2023). Biofilms are bacteria’s natural protection and can help them develop antibiotic resistance (Stewart, 2002; Tung et al., 2017). It has been observed that bacterial species capable of forming efficient biofilms are more resistant to drugs than those that do not form biofilms (Mirghani et al., 2022). The biofilm acts as a barrier to the entry of conventional antibiotics into cells and can be targeted as a drug target (Liu et al., 2024). It has been reported that disruption of the biofilm in *p*. *s aeruginosa* makes them more planktonic (Bukhari and Aleanizy, 2020). This implies that a drug targeting biofilm formation can be used as an antibiotic. Thus, the bacteria may lose resistance once the biofilm is disrupted after penetration and destroyed (Nadar, et al., 2022). Another novel aspect of developing new antibiotics is targeting quorum sensing (Tung et al., 2017). Bacteria use quorum sensing to communicate with one another. (Das et al., 2026). Green synthesis of ZnO NPs offers eco-friendly advantages and various biomedical applications.

In this study, ZnO NPs were green synthesized using an aqueous extract of lemon peel and comprehensively characterized using multiple techniques. The antibacterial and antibiofilm activities of the biosynthesized LP-ZnO particles were evaluated against six pathogenic Gram-positive and Gram-negative bacteria. Furthermore, an exploratory protein–protein interaction (PPI) network analysis was performed to investigate potential bacterial targets and pathways associated with major lemon peel phytochemicals, thereby providing complementary mechanistic insights into the possible contribution of phytochemical-associated interactions to the antibacterial activity of LP-ZnO nanoparticles.

## Materials and methods

### Materials

Zinc nitrate hexahydrate (Zn(NO_3_)_2_·6H_2_O), utilized as a metal precursor for the synthesis of ZnO nanoparticles, was procured from Sigma-Aldrich. Ammonium hydroxide (NH_4_OH) was purchased from Sigma-Aldrich. Dried lemons were purchased from the local market in Jeddah, Saudi Arabia. All solutions and plant extracts were produced using deionized water.

### Preparation of LP-ZnO NPs

**FIGURE 1:**
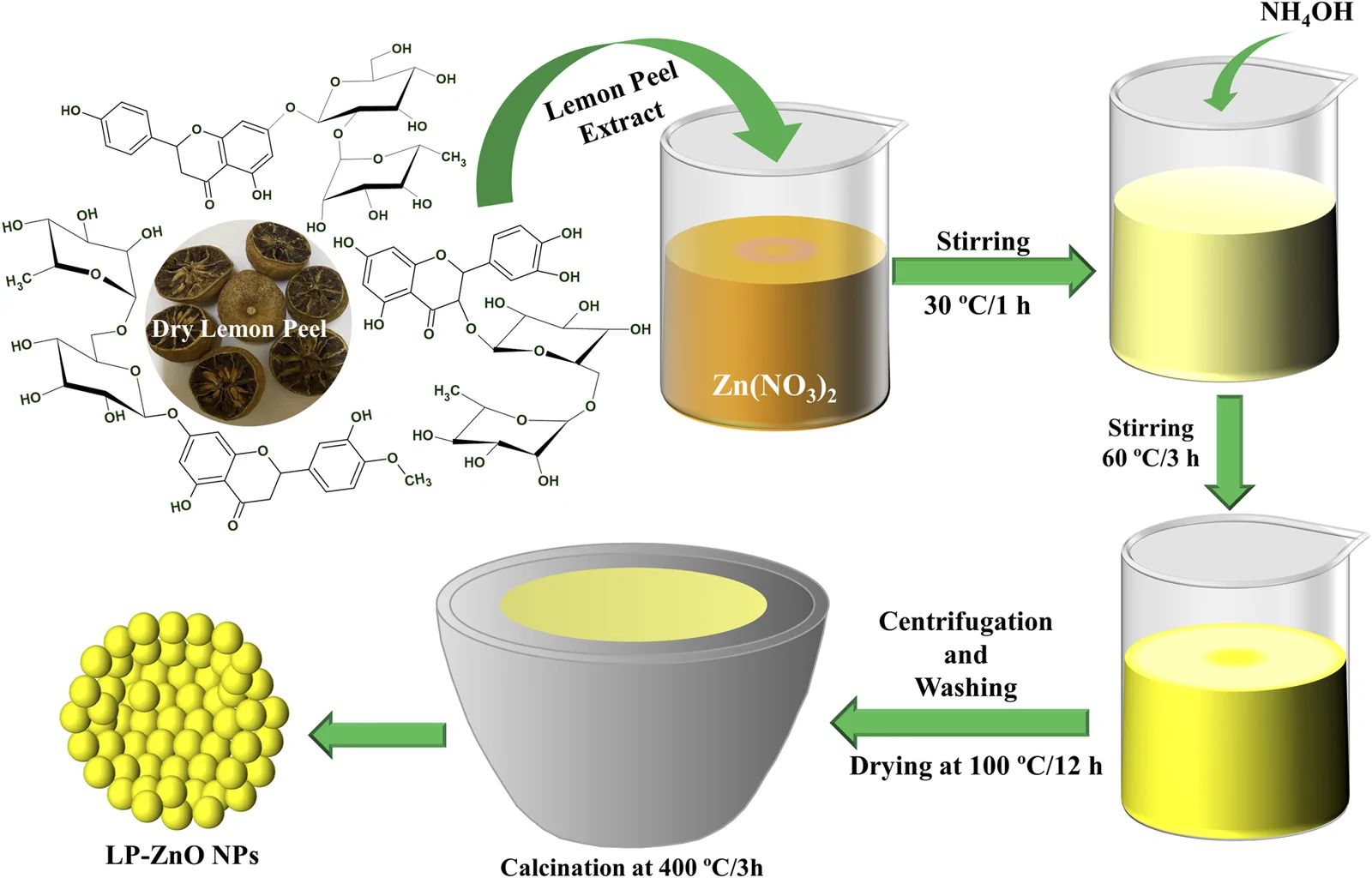
Schematic representation of LP-ZnO NPs synthesis.

To prepare the lemon peel extract, dried lemon peel was washed with tap water, then with deionized water, and then dried in an oven. After that, it was ground into a medium-fine powder. In a 250 mL beaker, 5 g of the powder was placed with 100 mL of deionized water and heated at 80 °C for 3 h. The extract was then filtered by vacuum filtration using Whatman No. 1 filter paper and stored in a refrigerator for further use. LP-ZnO NPs were prepared using a green approach, employing dried lemon peel aqueous extract in the precipitation method. In a typical experiment, 5 g of zinc nitrate was dissolved in 50 mL of distilled water, and 50 mL of aqueous lemon peel extract was added to this solution, with continuous stirring for 1 h at 30 °C. The pH of the reaction mixture was adjusted to 12 by adding NH_4_OH dropwise, and the mixture was stirred continuously for 3 h at 60 °C to ensure complete precipitation. After some time, the colour changed from light brown to light yellow, indicating that stabilized ZnO NPs have formed using lemon peel extract. The light-yellow precipitate was collected by centrifuging at 4000 rpm for 20 min. This light-yellow solid material was washed several times with a 3:2 mixture of distilled water and ethanol to eliminate all residual impurities, then dried in an oven at 100 °C for 12 h. The dried light-yellow powder was calcined at 400 °C for 3 h. The schematic representation of ZnO NPs synthesis is shown in Figure 1.

### Characterization of LP-ZnO NPs

The as-prepared ZnO NPs were characterized for their physicochemical properties, surface morphology, and thermal stability using different well-known analytical techniques. A Bruker D8 Advance diffractometer and CuKα radiation (λ = 1.5418 Å) produced at 40 kV and 30 mA current was used for X-ray diffraction (XRD) analysis, confirming the hexagonal wurtzite structure of the ZnO NPs by determining the crystallite phase, crystallite size, and structural purity. UV-DRS spectra of the ZnO NPs were obtained from 200 to 900 nm wavelength range using ultraviolet-diffused reflectance (UV-DRS) spectroscopy (Shimadzu, Japan) to determine the optical properties. Bruker ATRII FTIR analysis in the 4000-400 cm^-1^ region confirmed the active participation of functional groups in stabilization/capping and reduction during the preparation of ZnO NPs. To determine the surface morphology, elemental composition, and particle size, advanced electron microscopy techniques were used to provide detailed insights. Surface morphology and elemental composition characterization of the as-synthesized LP-ZnO NPs were conducted using a scanning electron microscope (SEM) FEI Nova Nano SEM 350 coupled with Energy Dispersive X-ray spectroscopy (EDX) EDS Bruker. The HR-TEM analysis was performed on FEI-Tecnai 200. Raman spectra were recorded at room temperature using a Bruker MultiRAM FT-Raman spectrometer. TGA was performed using a NETZSCH Thermogravimetric Analyzer STA 449 F3. Zeta potential measurements were performed using a Malvern Panalytical Zetasizer Pro (Malvern Panalytical Ltd., UK) based on Electrophoretic Light Scattering (ELS).

#### Bacterial strains and growth conditions

Six bacterial strains *E. Coli,* (ATCC 25922) *P. Aeruginosa* (ATCC 27853) *K. Pneumoniae, (*ATCC 13883) *S. typhimurium* (ATCC 13311) *S. Aureus,* (ATCC 25923) and *S. epidermidis* (ATCC 12228) used for this study and were cultured in nutrient broth, stored at −80 °C in 20% glycerol solution.

### Minimum inhibition concentration (MIC)

In accordance with CLSI procedures, a microbroth dilution test was performed to determine the MIC. The LP-ZnO nanoparticles were solubilized in 1% dimethyl sulfoxide (Sigma-Aldrich, Inc., St. Louis, MO, United States) to formulate a 100 μg/mL stock solution. The MIC for LP-ZnO NPs was determined by identifying the lowest concentration at which growth inhibition was evident. A 96-well plate was used for MIC assessment, with all bacterial strains incubated at 37 °C for 24 h. Further, 5 µL of the R7017 Resazurin sodium solution was added to the wells, and the results were recorded. Amoxicillin, a broad-spectrum antibiotic, served as the positive control, whilst 1% DMSO functioned as the negative control.

### Well diffusion assay

Antibacterial efficacy was evaluated using the agar well diffusion technique. Inoculation of 10^6^ CFU/mL bacterial strains was performed on Mueller–Hinton agar medium (MHA). A sterilized cork borer was employed to create five apertures in each culture plate. 100 µL of Amoxicillin was used as a positive control, while sterile distilled water (SDW) was used as a negative control. The last three holes were administered LP-ZnONPs at concentrations of 25, 50, and 75 μg/mL. The plates were incubated at 37 °C for 24 h. The average standard deviation (SD) of triplicate experiments indicated the zone of inhibition around the wells.

### Disk diffusion method

A further disc diffusion assay was conducted to verify the antibacterial efficacy of the synthesized LP-ZnO nanoparticles. Bacterial strains (10^6^ colony-forming units/mL) were inoculated onto Mueller-Hinton agar. The LP-ZnONPs were allowed to diffuse into the agar at ambient temperature for 30 min after deposition onto a 7 mm paper filter disc at three concentrations (25, 50, and 75 ug/mL). Amoxicillin and sterile distilled water were used as positive and negative controls. The plates were subsequently incubated at 37 °C for 24 h. The inhibitory zone was measured in triplicate, and the mean standard deviation was recorded.

### Biofilm inhibition assay

The antibiofilm activity of LP-ZnO NPs was determined using the crystal violet method in a flat-bottom 96-well microtiter plate. At 30 °C for 24 h, all the bacteria were cultured in nutrient broth with 25 μg/mL or 50 μg/mL LP-ZnO NPs. To remove cells from microplates after incubation, they were rinsed twice with phosphate-buffered saline (pH 7.4). The biofilm was stained with a 0.1% (200 L) solution of crystal violet after 15 min. Before PBS was used again, the wells were cleaned twice with 95% ethanol (200 L). This biofilm production was measured quantitatively using an iMark microplate reader (Bio-Rad Laboratories, ArIrvine, CA, United States) at OD570 nm.

### Identification of pectin, hesperidin, and eriocitrin target genes

The saturated data files (SDFs), encompassing the chemical structures of pectin, hesperidin, and eriocitrin, were retrieved from the PubChem database (https://pubchem.ncbi.nlm.nih.gov). These files were then uploaded to PharmMapper (Liu et al., 2010) webserver for the prediction of target genes for each of these compounds. These phytochemicals were selected because they represent major constituents of lemon peel extract and are reported to participate in the reduction, stabilization, and surface capping of biosynthesized ZnO nanoparticles.

### Protein-protein interaction network construction

Putative *E*. *coli* protein targets for three compounds (pectin, hesperidin, and eriocitrin) were compiled from the candidate lists. To obtain a single, non-redundant input list for network analysis, duplicate entries and non-*E. coli* records were removed, yielding 84 unique query proteins. The 84 non-redundant *E. coli* target proteins were submitted to the STRING database (STRING v12.0; https://string-db.org/) to retrieve protein–protein interactions (PPI). The STRING search included experimentally supported interactions and those from curated databases; the minimum required interaction score was set to 0.70 (high confidence). In the STRING query, we allowed up to 50 first-shell interactors to provide functional context for the input targets. Furthermore, the functional enrichment (GO Biological Process and KEGG pathway enrichment) was also performed by STRING using the full *E. coli* background. Statistical significance was assessed via FDR-adjusted p-values (Benjamini–Hochberg correction); terms with FDR < 0.05 were considered significant. Full enrichment tables and matching protein lists produced by STRING are provided in the Supplementary Tables S1–S3; the TSV files you uploaded).

#### Statistical analysis

All experiments related to antibacterial and antibiofilm activity were performed in triplicate, and the results are presented as the mean ± standard deviation (SD). The statistical analysis was performed using various software, including Excel and Origin.

## Results and discussion

**FIGURE 2:**
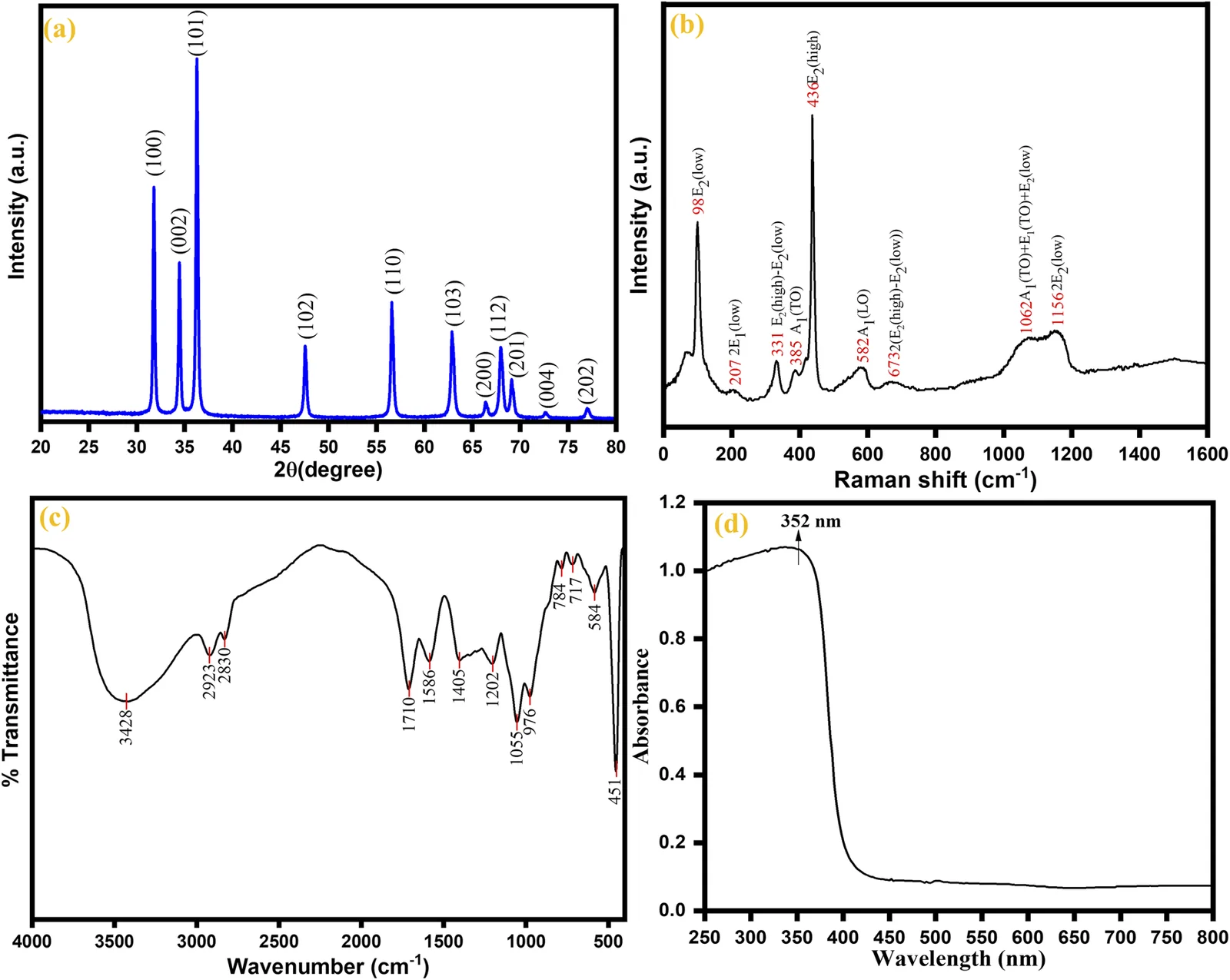
**(a)** X-ray diffraction pattern, **(b)** Raman spectra, **(c)** FTIR, and **(d)** UV-Vis DRS spectra of LP-ZnO NPs.

XRD was used to investigate the production of the LP-ZnO NPs stabilized by lemon peel extract. The analysis was derived from the investigation of the phase geometry of the biosynthesized ZnO NPs. Further, the crystalline nature of such nanoparticles is determined by their profile, as shown in Figure 2a. The observed narrow, sharp diffraction peaks are from bio-synthesized, stable, and highly pure crystalline ZnO NPs. The X-ray diffraction patterns has revealed prominent peaks at 2θ values: 31.86°, 34.48°, 36.41°, 47.65°, 56.68°, 62.96°, 66.41°, 67.93°, 69.10°, 72.55°, and 77.10°, corresponding to the (100), (002) (101), (110) (103), (200) (112), (201) and (202) crystal planes. The results obtained are the characteristic crystal planes of the hexagonal wurtzite structure in biosynthesized ZnO NPs and are supported by literature reports, as per JCPDS-361451 (Sapkota et al., 2019). Meanwhile, upon recording the FHWFM of the diffraction peaks, the average crystallite size of the LP-ZnO NPs was calculated using the Debye-Sheerer equation D = Kλ/βcosθ, where D represents the size of a crystallite, K corresponds to Sheerer constant = 0.94, which varies depending on the particle shape (hexagonal particles have a value of 0.90 for this factor), λ is the wavelength of X-rays, CuK radiation (0.15406 nm), β is the FHWFM (Full width at half maximum) in radians (β = 0.47078, as are numerical valued for the most of the intense radiations). After putting these values in the equation, the as-synthesized LP-ZnO NPs had an average crystalline size of ≈ 20 nm.

Meanwhile, Raman spectroscopy was performed on the biosynthesized ZnO NPs at room temperature, as shown in Figure 2b. The distinct intensity peaks are observed at positions near 98, 207, 331, 385, 436, 582, 673, 1062, and 1156 cm^-1^. The intense peak at 98 cm^-1^ has been attributed to the hexagonal crystal structure of the ZnO complex’s E2 (Low), whereas the peak at 207 cm^-1^arises after the 2E1 (low) second-order phonon mode. Besides, ZnO oxygen vacancies are associated with the peak at 582 cm^-1^ corresponding to A1 (LO). In contrast, the other two peaks at 1062 and 1156 cm^-1^ are attributable to A1 (TO)+E1 (TO)+E2 (low) combinations at the A and H points, respectively, and to 2E2 (low). In addition, the appearance of the E2 (high) mode in the annealed ZnO NP samples signifies improved crystalline quality and structural order in the synthesized material (Figure 2b). The presence of a high-intensity peak at 436 cm^-1^ is attributed to local vibrational modes associated with the disappearance of intrinsic lattice defects, thereby indicating that the heat treatment improved the overall crystalline quality of the ZnO nanopowders. Furthermore, these findings support the XRD results, indicating the formation of crystalline ZnO upon annealing. The FTIR spectra of the LP-ZnO NPs shown in Figure 2c reveal major characteristic peaks corresponding to the OH, C-H, C-O, C=C, and C=O stretching. The peak at 3428 cm^-1^ is assigned to the stretching and bending vibrations of the OH groups. The absorption peaks at 2923 cm^-1^ and 2830 cm^-1^ are assigned to the C-H stretching. The bands at 1710 cm^-1^ and 1586 cm^-1^ correspond to the C=O and C=C stretching vibrations, respectively. The peaks at 1405 cm^-1^ and 1055 cm^-1^ correspond to the bending vibration of C-H and C-O functional groups, respectively. The absorption peak at 1202 cm^-1^ corresponds to the bending vibration of C-O stretching of residual organic constituents. The band at 976 cm^-1^ corresponds to the C-O stretching vibration due to the presence of organic residues on the surface of nanoparticles. Additionally, the peaks at 784 cm^-1^ and 717 cm^-1^ correspond to the Zn-O lattice stretching vibration, which proves the successful formation of ZnO nanoparticles. The signals at 584 cm^-1^ and 541 cm^-1^ are fingerprint bands corresponding to the stretching vibration of the Zn-O bond and confirm the ZnO nanoparticle formation.

UV-visible Diffuse Reflectance Spectroscopy (UV-Vis DRS) measurements were used to determine the optical properties of the LP-ZnO NPs as illustrated in Figure 2d. The UV-Vis DRS spectrum displays a well-defined characteristic absorption edge in the ultraviolet region (λ_max_ = 352 nm), a distinctive feature of ZnO nanoparticles that confirms their semiconducting properties (Menazea et al., 2021; Sharma et al., 2025). The appearance of the absorption edge in the UV region arises due to the electron excitation from the valence band composed of 2p orbitals of Oxygen to the conduction band composed of 4 s orbitals of zinc. It is well established from the observed results that the successful synthesis of ZnO NPs with the characteristic electronic structure of crystalline nature is confirmed by the distinct absorption edge. In addition, this distinct absorption edge in the UV region confirms high photoresponse, which is desirable for optoelectronic, photocatalytic, and antimicrobial applications.

**FIGURE 3:**
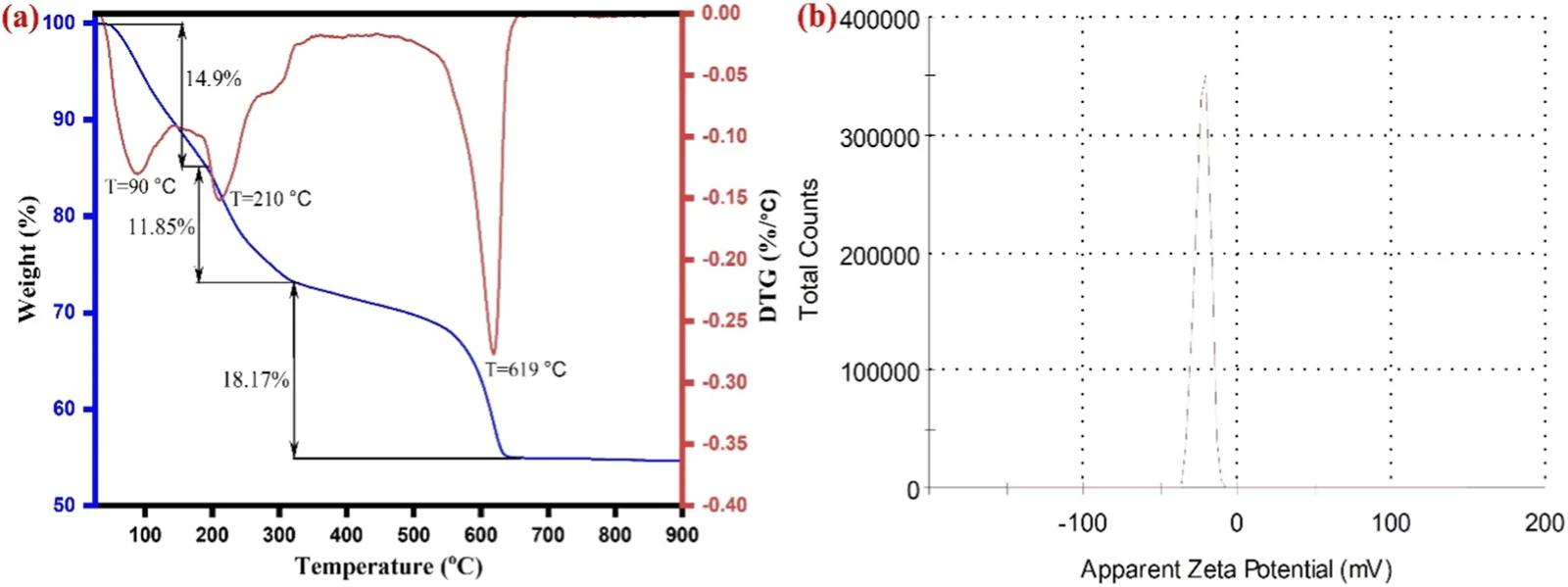
**(a)** TGA/DTG and **(b)** Zeta potential curves of LP-ZnO NPs.

Thermogravimetric Analysis (TGA) and derivative Thermogravimetric Analysis (DTG) of biosynthesized LP-ZnO NPs were performed under controlled conditions to assess thermal behavior and weight-loss percentage, as shown in Figure 3. Three distinct weight-loss stages, namely the removal of residual moisture and physically surface-adsorbed water molecules, the decomposition of surface-bound phytochemicals, and the thermal stabilization of the as-prepared nanoparticles, are usually observed in the TGA survey. Figure 3 shows the TGA/DTG curve at a heating rate of 10 °C/min over a temperature range of 25 °C to 900 °C. The results revealed that calcined LP-ZnO NPs exhibit a first weight loss of 14.90% between 50 °C and 190 °C. This weight loss is attributed to the removal of physically adsorbed water molecules by evaporation and is accompanied by a weak DTG peak, as shown in the DTG curve in Figure 3 (Cao et al., 2020; Khaled et al., 2026). This DTG peak is attributed to the removal of chemisorbed and physically adsorbed water on the surface of the biosynthesized LP-ZnO NPs. The second observed weight loss was approximately 11.85% in the temperature range of 190 °C–320 °C, attributed to the thermal decomposition of the phytochemicals present in the lemon peel extract used to synthesize ZnO nanoparticles. A well-defined DTG peak was observed in this temperature range, corresponding to the rapid thermal degradation of phytochemicals, including flavonoids, polyphenols, and other biomolecules (Cao et al., 2020; Khaled et al., 2026). These phytochemicals play a significant role as stabilizing agents in the green synthesis of LP-ZnO NPs. The final weight loss of 18.17% was observed in the temperature range of 320 °C to 640 °C, which corresponds to the removal of remaining strongly bound organic constituents at the surface of ZnO NPs, confirms the final crystallization, and thermal stability of the as-prepared LP-ZnO NPs. In this temperature range, a DTG curve was observed, typically associated with complex reactions that contribute to the formation of thermally stable, crystalline ZnO NPs. The TGA curve reveals that above 640 °C, no further weight loss was observed, which indicates that as-prepared LP-ZnO NPs become a thermally stable structure (Cao et al., 2020; Khaled et al., 2026). The material was found to be thermally stable up to 640 °C, with a total weight loss of 44.92%.

The Zeta potential analysis was performed to check the stability and surface charge of the biosynthesized LP-ZnO NPs. Figure 3b represents the Zeta potential curve of LP-ZnO NPs. The average Zeta potential value of LP-ZnO NPs synthesized by using aqueous lemon peel extract was found to be −22.4 mV (Faisal et al., 2021). The sharp and narrow peak observed in the Zeta potential curve represents the uniform charge distribution of the LP-ZnO NPs. The observed negative surface charge, indicating strong anionic character, is due to the binding affinity of the phytochemicals for the as-prepared nanoparticles, thereby enhancing the stability of ZnO NPs and reducing particle aggregation. The presence of the surface functional groups (e.g., -OH, COOH) of the phytochemicals, including polyphenols, flavonoids, and other organic constituents, offers electrostatic repulsion between ZnO NPs and results in the restriction of particle aggregation, thereby enhancing the colloidal stability (Sowbarnika et al., 2026).

### Morphological analysis

**FIGURE 4:**
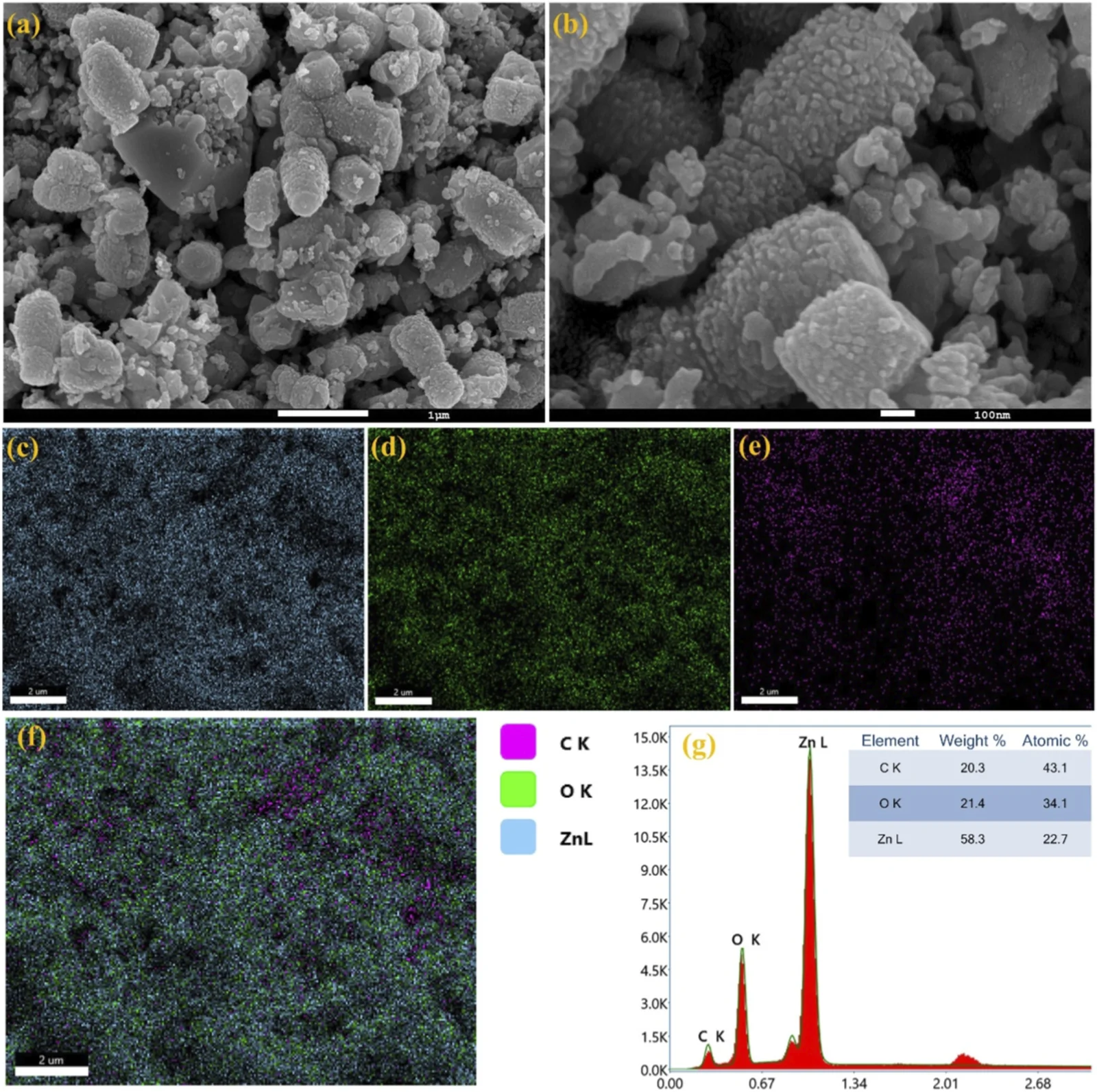
SEM images at **(a)** 100 nm scale and **(b)** 1 µm scale, elemental mapping of **(c)** Zn, **(d)** O, **(e)** C, **(f)** ZnO, and **(g)** EDX spectra of ZnO NPs.

The surface morphology of as-synthesized LP-ZnO NPs was characterized by SEM, and element composition analysis was achieved by EDX analysis, as shown in Figure 4. The SEM analysis was conducted at 100 nm and 1 µm scales to examine the surface topography and morphology of the LP-ZnO NPs. The SEM micrograph of the LP-ZnO NPs at a 1 µm scale shows irregularly shaped particles and spherical and near-spherical particles that have clumped together in a mushroom pattern (Figure 4a). However, at the 100 nm scale, the SEM image of LP-ZnO NPs showed nanoparticle aggregation, with spherical particles clumping into a fused pattern (Figure 4b). On the other hand, the presence of major constituent elements (Zn and O) in biosynthesized LP-ZnO NPs was confirmed from the EDX spectrum, which confirms the elemental purity and successful formation of pure ZnO NPs. The corresponding elemental mapping of the individual elements (Zn and O) and the ZnO NPs is shown in Figures 4c–f. The corresponding weight percentages of the elemental composition of zinc (58.3%), oxygen (21.4%), and carbon (20.3%) further certify the biosynthesis of LP-ZnO NPs (Figure 4g). The high carbon signal in LP-ZnO NPs may be due to the surface-bound organic constituents that remain intact even after calcination, as observed in the TGA analysis of the calcined ZnO NPs. TGA analysis indicated that most of the organic constituents decomposed during calcination, suggesting that the detected carbon does not indicate incomplete conversion of the precursor to ZnO NPs. The surface-bound phytochemicals present on the surface of the ZnO NPs could influence the antibacterial properties through improved interaction between the nanoparticles and the cell membrane and enhanced dispersion, which, as a result, contributes synergistically to antibacterial activity. Demissie et al. (2020) reported spherical and nanorod-shaped morphology of ZnO NPs as synthesized from “*Lippiaadoensis*”. Furthermore, Putta and Vastrad reported that ZnO NPs with spherical morphology were biosynthesized from citrus peel extract (Putta and Vastrad, 2023).

**FIGURE 5:**
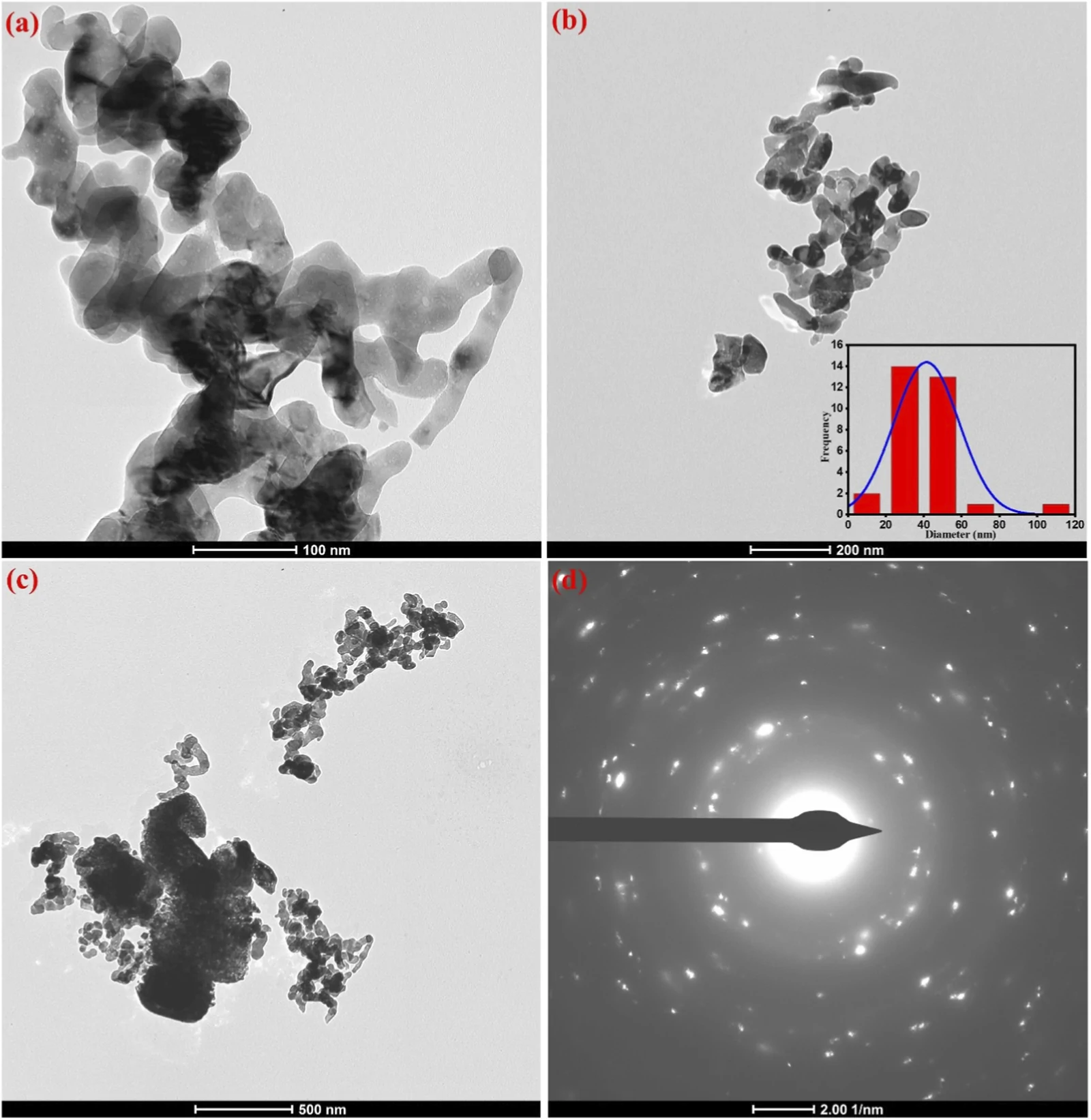
**(a–c)** TEM images at 100 nm, 200 nm, and 500 nm nano scales and **(d)** SAED pattern of bio-synthesis of LP-ZnO NPs.

TEM analysis was performed to determine the morphology, particle size, physical dimensions, and dispersibility of the as-prepared LP-ZnO NPs (Volochaev and Tsaturyan, 2026), and the corresponding micrographs are shown in Figures 5a–c at 100 nm, 200 nm, and 500 nm scales, respectively. The particle size of LP-ZnO NPs was determined to be ∼41 nm. Further, the particle size distribution histogram of LP-ZnO NPs shown in Figure 5b (inset) confirmed the narrow size distribution of the as-prepared NPs. The TEM images confirm the formation of nearly spherical and polygonal ZnO NPs, with a few irregular particles. The TEM micrographs exhibit moderate agglomeration, which is commonly observed in biosynthesized nanoparticles. Furthermore, selected area electron diffraction (SAED) is a crystallographic technique used to determine the crystal structure of nanoparticles. The SAED pattern of LP-ZnO NPs exhibits bright concentric diffraction rings or spots indexed to specific crystal planes, specifically (100), (002) (101), (102) (110), (103), and (112) planes (Figure 5d). These observations confirm the hexagonal wurtzite structure of ZnO and the high crystallinity of biosynthesized LP-ZnO NPs.

### The plausible mechanism involved in the formation of LP-ZnO NPs

There is a need for further research into the mechanisms underlying phytochemical-mediated synthesis of metal oxide nanoparticles. Proteins, carbohydrates, amino acids, tannins, steroids, and cardiac glycosides are abundant in dried lemon peel extract, along with flavonoids, phenols, alkaloids, and proteins. Due to their reducing and stabilizing/capping characteristics, the phytochemicals present in the aqueous extract are primarily responsible for the formation of metal and metal oxide nanoparticles. Based on prior research, we propose a possible mechanism by which the phytochemicals in lemon peel extract interact with Zn^2+^ ions to form a metal-ligand complex that regulates nucleation and particle growth during the synthesis of ZnO NPs. On the addition of NH_4_OH and subsequent hydrolysis, zinc hydroxide (Zn(OH)_2_) nuclei are formed as an intermediate. The Zn(OH)_2_ intermediates undergo a condensation process and grow into nanoparticles. Additionally, phytochemicals aid in the formation and stabilization of metal oxide nanoparticles, thereby preventing particle aggregation. To begin, the polyphenolic molecules in the plant extract undergo deprotonation at their hydroxyl groups, followed by binding of the deprotonated polyphenols to zinc ions via complexation (Nava et al., 2017). Thermal breakdown is the final process for obtaining ZnO nanoparticles. The most active functional groups in phytochemicals are hydroxyl groups, which enable Zn-ion complexation, hydrolysis, and thermal decomposition, thereby forming ZnO nanoparticles. The results of this investigation revealed the existence of polyphenol compounds as stabilizing and capping agents that contributed to the production of ZnO NPs. Moghaddam et al. (2017) proposed a mechanism for the green synthesis of ZnO NPs through the interaction between zinc ions and amino acid hydroxyl groups. By regulating particle growth and reducing particle aggregation, the released amino acid also helped stabilize ZnO NPs. The ZnO nanoparticles showed less aggregation when Azadirachta indica leaves were used in the synthesis process, further confirming the role of phytochemicals in stabilizing and controlling the morphology of the ZnO NPs (Da’na et al., 2021).

### Biological activity of LP-ZnO NPs

#### Minimum inhibitory concentration (MIC)

The antibacterial activity of LP-ZnO NPs was determined against four Gram-negative bacterial species *(E. coli, P. Aeruginosa, K. Pneumoniae, S. typhimurium) and two species of Gram-positive* bacteria *(S. Aureus* and *S. epidermidis*). The comparative antibacterial effectiveness of LP-ZnO nanoparticles against various bacterial strains was assessed by a well-diffusion assay and quantitatively via a disk-diffusion assay.

The MIC of LP-ZnO NPs was recorded as 5 μg/mL against *S. epidermidis,* followed by *S. Aureus, P. aeruginosa, S. typhimurium, K. Pneumoniae, and E. coli,* at 7, 11, 14, 14,15 μg/mL, respectively*,* whereas the MIC average of amoxicillin was 9 μg/mL) against all the bacterial strains in Table 1.

**TABLE 1:** Minimum inhibitory concentration (MIC) of bacterial strains when treated with LP-ZnO NPs and Amoxicillin.

Strains	LP-ZnO NPs MIC (µg/mL)	Amoxicillin MIC (µg/mL)
*E. Coli*	15	9
*K. Pneumoniae*	14	9
*P. Aeruginosa*	11	9
*S. Typhimurium*	14	9
*S. Aureus*	7	9
*S. Epidermidis*	5	9

The minimum bactericidal concentration (MBC) of LP-ZnO nanoparticles exceeded the minimum inhibitory concentration (MIC), signifying that LP-ZnO nanoparticles can suppress bacterial proliferation at elevated doses (Table 2).

**TABLE 2:** Minimum bactericidal concentration (MBC) of bacterial strains when treated with LP-ZnO NPs and Amoxicillin.

Strains	LP-ZnO NPs (µg/mL) (MBC)	Amoxicillin (µg/mL) (MBC)
*E. Coli*	28	15
*K. Pneumoniae*	25	15
*P. Aeruginosa*	20	15
*S. Typhimurium*	25	15
*S. Aureus*	16	15
*S. Epidermidis*	9	15

Previous studies on the antibacterial efficacy of ZnO nanoparticles against non-resistant Gram-negative bacterial strains reported an inhibitory dose of 0.015 mg/mL for *E. coli* and *S. typhimurium*, whereas a minimum dose of 0.005 mg/mL was sufficient to inactivate *K. pneumonia* (Brayner et al., 2006; Siddiqi et al., 2018). Similarly, our MIC results confirmed that LP-ZnO NPs can inhibit the growth of Gram-negative bacteria; however, they are more effective against Gram-positive bacteria. Further, it has been reported that ZnO nanoparticles can inhibit the growth of *S*. *epidermidis*, a Gram-positive bacterium; the minimum inhibitory concentration of NPs is 25 μg/mL (Palanikumar et al., 2014). Our MIC results also confirmed that LP-ZnO NPs can inhibit the growth of *S*. *epidermidis* at as low as 5 μg/mL. The results are consistent with our previous studies, in which synthesized ZnO NPs showed activity against the opportunistic pathogen *Chromobacterium violaceum* (Kamli et al., 2021).

#### Antibacterial activity of LP-ZnO NPs using agar diffusion assay

**FIGURE 6:**
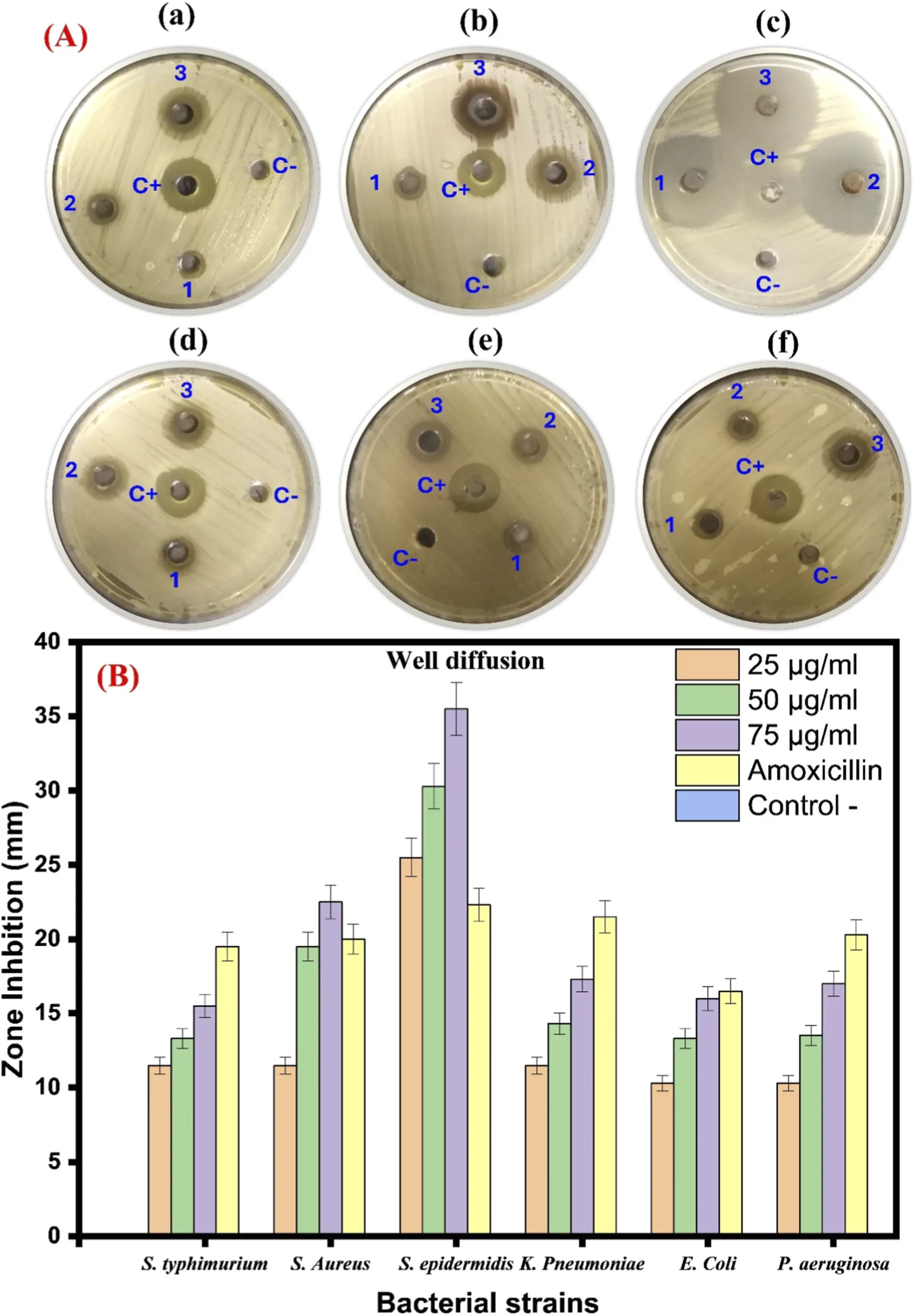
**(A,B)** Inhibition zone (mm) of LP-ZnO NPs by agar Well diffusion method against bacteria tested. a; *S*. *typhimurium*, b; *S. Aureus*, C; *S. epidermidis*, D; *K. Pneumoniae,* E; *E. Coli*, F; *P. aeruginosa*, 1; 25 μg/mL, 2; 50 μg/mL, 3; 75 μg/mL, C+; Amoxicillin, C-; SDW.

A well-diffusion assay was conducted using various bacterial strains; a distinct zone surrounding the treated LP-ZnO nanoparticles indicated that these nanoparticles exhibit antibacterial activity, inhibiting the growth of Gram-negative and Gram-positive bacteria. The findings indicate that the inhibition zones range from 10.3 ± 0.5 mm to 35 ± 0.5 mm, as depicted in Figure 6. The most pronounced antibacterial activity was shown against *S. epidermidis* (35 mm), followed by *S. aureus* and *K. pneumoniae* (22.5 mm and 17.3 mm, respectively). The minimal inhibitory zone observed was 16 mm against *E. coli* at a dose of 75 μg/mL across all experiments. We also noted the impact of amoxicillin (16.5–22.3 mm) against all employed bacterial pathogens (Figures 6A,B). Previous studies suggest that green synthesis of zinc oxide nanoparticles from Pomegranate extracts exhibits dose-dependent antibacterial activity. This study demonstrated that increasing nanoparticle concentration enhanced their antibacterial activity. A possible reason may be the synergistic effect between oxide nanoparticles and Pomegranate extracts (Ifeanyichukwu et al., 2020). Similarly, Bizuayehu et al. reported that zinc oxide nanoparticles synthesized using Rumex Nervosus Vahl leaf extract showed antibacterial activity against *Bacillus*, *S. aureus*, *E. coli*, and *K. pneumoniae* (Bizuayehu et al., 2025).

### Disk diffusion assay

**FIGURE 7:**
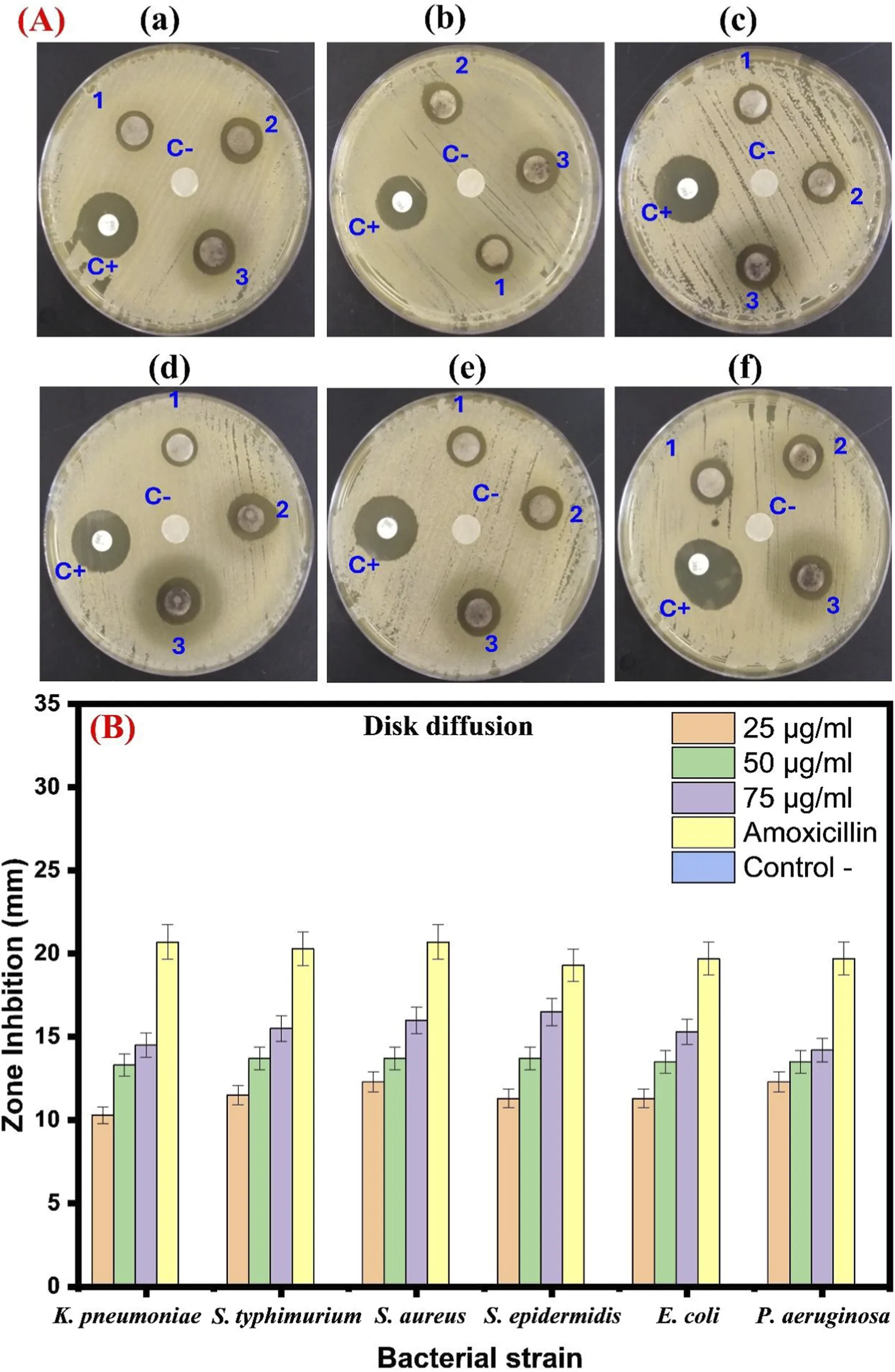
**(A,B)** Inhibition zone (mm) of LP-ZnO NPs by agar well diffusion method against bacterial tested. A; *K. Pneumoniae*, B; *S. typhimurium*, C; *S. Aure*us, D; *S. epidermidis,* E; *E. Coli*, F; *P. aeruginosa*, 1; 25 μg/mL, 2; 50 μg/mL, 3; 75 μg/mL, C+; Amoxicillin, C-; SDW.

Results from the disk diffusion assay showed a clear zone around the disk, indicating that the LP-ZnO NPs exhibited antibacterial activity by inhibiting the growth of the bacterial strains (Figures 7A,B). The Disk-diffusion assay exhibits the highest antibacterial efficacy against *S. epidermidis* (16.5 mm), followed by *S. Aureus* (16 mm), then *S. Typhimurium*, *E. coli*, and *K. Pneumoniae*, and *P. Aeruginosa* (15.5, 15, 14.5 and 14.2 mm, respectively) at 75 μg/mL for all assays. Amoxicillin 10 μg/mL was used as a positive control, and its effect was between (19.3–20.7 mm) against all bacterial strains. Comparable outcomes were documented in a prior investigation utilizing disk diffusion assays, indicating that the green synthesis of zinc oxide nanoparticles using Phlomis leaf extract can impede the growth of *S. aureus* and *E. coli* (Alyamani et al., 2021). Moreover, a prior study indicated that green synthesis of ZnONPs using the essential oil of *Bunium persicum* exhibited antibacterial activity (Akhtari et al., 2025).

The biogenesis of ZnO NPs by using aqueous leaf and seed extracts of Croton macrostachyus Hochst. ex Delile, as reducing and stabilizing reagents, showed high antibacterial efficacy against *S. aureus*, *P. aeruginosa*, and *K. pneumoniae*. Further, they reported that crude plant extracts of Croton macrostachyus showed lower antibacterial efficacy than ZnO NPs synthesized from these extracts (Habtemariam et al., 2026). In another study by Gupta et al., ZnO NPs were synthesized using leaf extract of Catharanthus roseus (C. roseus). The results showed significant antibacterial activity of ZnO NPs against S. aureus, S. pyogenes, B. cereus, *P. aeruginosa*, *P. mirabilis*) and *E. coli* (Gupta et al., 2018). Thus, previous studies indicate that the synthesis of ZnO NPs using a plant extract confers antibacterial activity; a possible reason may be the synergistic effect between ZnO and the plant extract.

### Biofilm inhibition by LP-ZnO NPs

**FIGURE 8:**
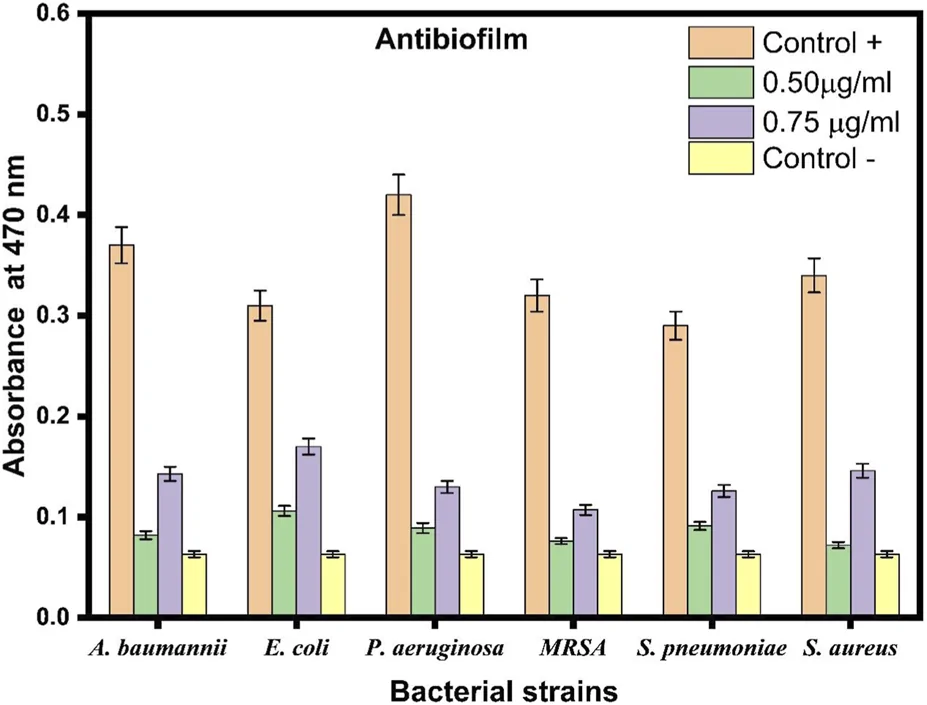
Antibiofilm activity of LP-ZnO NPs against bacterial pathogens.

By observing the binding of 0.1% crystal violet to the biofilm formed by the bacterial cells in a microtiter plate, the anti-biofilm activity was examined *in vitro*. The measured optical density indicated these pathogens’ propensity to form biofilm. Using a microplate reader assay, the biofilm inhibition property of LP-ZnO NPs against different pathogenic strains *S. epidermidis, S. aureus, K. pneumoniae, P. aeruginosa, S. typhimurium*, and *E. coli* (Figure 8). The findings revealed that anti-biofilm activity increased with increasing nanoparticle concentration. After treating bacterial strains with LP-ZnO NPs at 50 and 75 μg/mL, the results showed a reduction in OD. Biofilm formation has caused significant concern and panic in clinical settings and has been directly linked to drug resistance (Liu et al., 2024; Singh et al., 2025). Different types of nanoparticles have also been reported to inhibit biofilm formation in various bacterial species. For example, in a study by Mohanta et al., synthesized silver nanoparticles were reported to possess antibacterial and anti-biofilm activities against *P. aeruginosa*, *E. coli*, and *S. aureus* (Mohanta et al., 2020). Previous studies have also reported that ZnO nanoparticles synthesized from Clitoria ternatea can serve as an effective alternative therapy for biofilm-associated oral infections (Lahiri et al., 2022). Similarly, another study found that zinc oxide nanospikes inhibited biofilm growth in *P. aeruginosa*. Consequently, it may be utilized to address drug-resistant *P. aeruginosa* infections (Khan et al., 2020). These biofilms allow bacteria to thrive in hostile environments and to resist antibiotics, making them hazardous. Biofilm-related infections: bridging the gap between clinical management and fundamental aspects of recalcitrance toward antibiotics (Lebeaux et al., 2014). Therefore, biofilms have amplified the challenge of using conventional antibiotics to suppress the infection altogether (Rasko et al., 2008).

### Bacterial target identification

Because phytochemicals present in lemon peel extract participate in nanoparticle formation and may remain adsorbed on the nanoparticle surface as capping agents, we explored their potential bacterial targets to obtain preliminary mechanistic insights into possible phytochemical-associated antibacterial effects. PharmMapper analysis was used to predict potential protein targets for the three compounds eriocitrin, hesperidin, and pectin, which are the main constituents of lemon peel (Zapata et al., 2026). The top 300 ranked hits for each compound were retrieved and filtered to retain only bacterial proteins (Supplementary Tables S1–S3). Among bacterial species, *E*. *coli* strain K12 was the most represented organism across all three compounds, yielding 29, 30, and 46 putative targets for eriocitrin, hesperidin, and pectin, respectively. Other bacterial species with notable representation included *B*. *subtilis* (9, 7, and 7 targets), *M*. *tuberculosis* (strain ATCC 25618: 3, 2, and 8 targets; strain CDC 1551: 3, 1, and 8 targets), *T*. *maritima* (4, 4, and 3 targets), *P*. *aeruginosa* (2, 5, and 2 targets), *G*. *stearothermophilus* (3, 0, and 4 targets), *Vibrio cholerae* (2, 3, and 2 targets), and *B*. *cereus* (1, 2, and 3 targets). Additionally, predicted targets were also observed for *K*. *pneumoniae* (3, 2, and 1 targets), *S*. *typhimurium* (2, 2, and 1 targets), *S*. *flexneri* (3, 0, and 1 targets), and *T*. *thermophilus* (1, 2, and 1 targets).

When compared to the bacterial strains employed in the experimental assays—*E*. *coli*, *P*. *aeruginosa*, *K*. *pneumoniae*, *S*. *typhimurium*, *S*. *aureus*, and *S*. *epidermidis*—at least one or more putative protein targets were identified for each compound among these species (Supplementary Tables S1–S3). Overall, these findings emphasize that *E. coli* is the most abundant predicted target host across all compounds, followed by *B. subtilis*, *M. tuberculosis*, and *T. maritima*. The broad distribution of predicted bacterial targets suggests that eriocitrin, hesperidin, and pectin have the potential to interact with bacterial proteins across Gram-negative and Gram-positive species.

### Protein–protein interaction (PPI) network analysis

**FIGURE 9:**
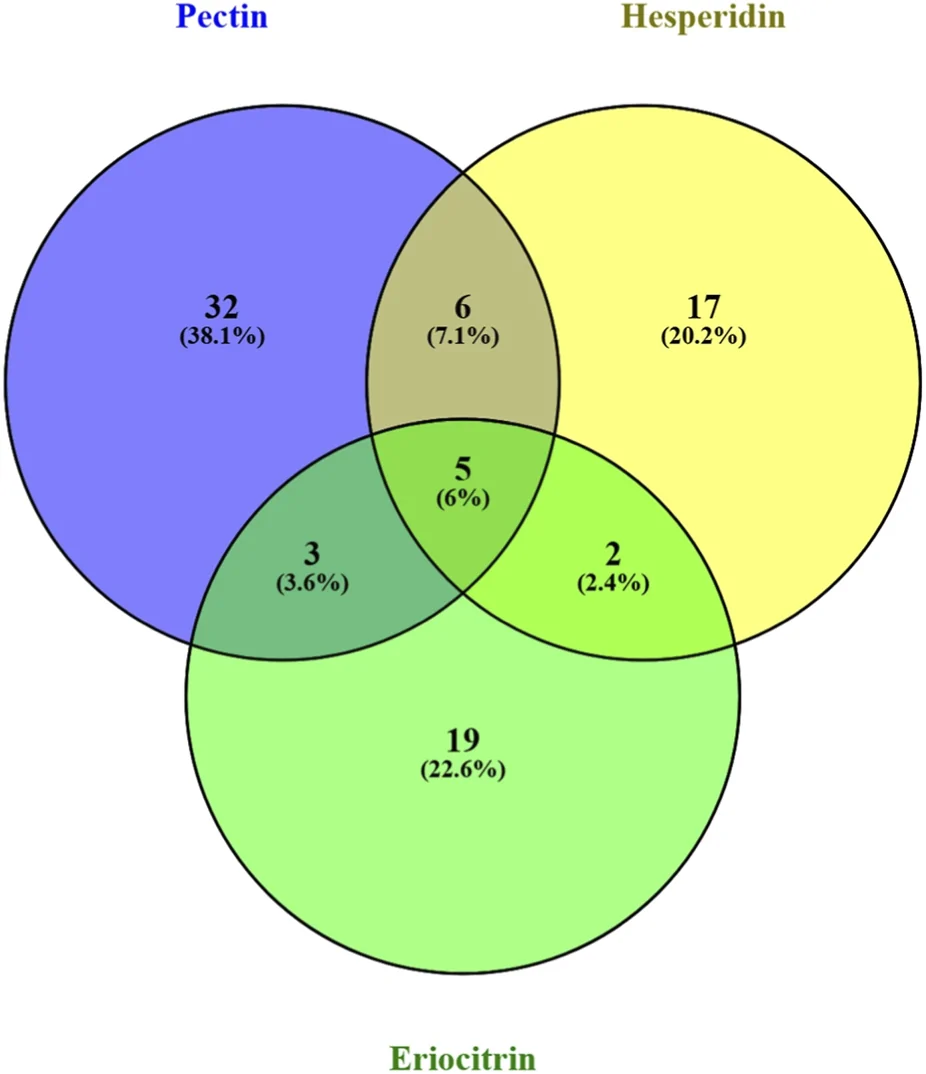
Venn diagram illustrating the distribution and overlap of predicted *E. coli* protein targets for pectin, hesperidin, and eriocitrin. The diagram highlights both compound-specific and shared targets, with five proteins common to all three compounds, indicating potential convergent molecular interactions.

To gain deeper insights into the potential biological relevance and functional relationships among the predicted targets, a protein–protein interaction (PPI) network analysis was performed. Since *E*. *coli* emerged as the most represented bacterial species across all three compounds (Supplementary Tables S1–S3), yielding substantially more predicted targets than any other bacterial species, it was selected as a model organism for network-based analysis. The larger target set enabled statistically robust PPI and enrichment analyses, thereby providing a comprehensive systems-level view of potential antibacterial mechanisms associated with phytochemicals. Firstly, a non-redundant set of 84 unique *E. coli* protein targets, compiled from the combined lists of all three compounds (29, 30, and 46), was used to generate an integrated PPI network. Comparison among compound-specific target sets revealed that five proteins—thymidylate synthase (ThyA), arabinose operon regulatory protein (AraC), D-galactose/methyl-galactoside binding periplasmic protein (MglB), cystathionine γ-synthase (MetB), and purine nucleoside phosphorylase (DeoD) were common to all three compounds (Figure 9).

These shared proteins were considered high-priority targets because they represent convergent predictions across chemically distinct phytochemicals. Functionally, these proteins are involved in essential bacterial processes, including nucleotide biosynthesis and salvage (ThyA and DeoD), methionine biosynthesis (MetB), carbohydrate uptake and utilization (MglB), and transcriptional regulation of carbon metabolism (AraC). The convergence of all three compounds on these proteins suggests that perturbation of nucleotide metabolism and carbon utilization may represent common antibacterial mechanisms associated with lemon peel-derived phytochemicals. Collectively, these findings indicate convergent molecular recognition in *E. coli* despite the distinct chemical scaffolds of eriocitrin, hesperidin, and pectin.

**FIGURE 10:**
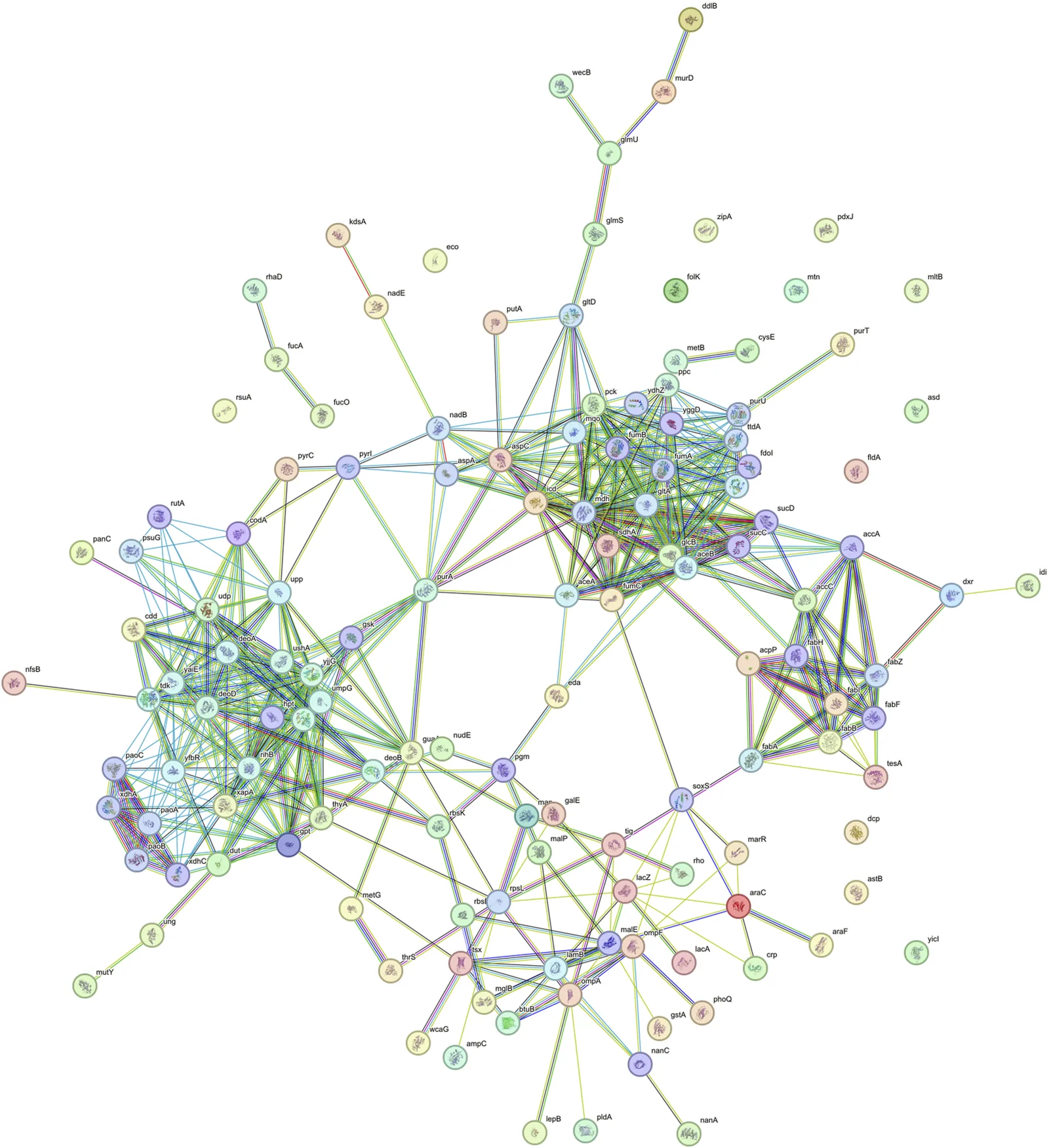
Network representation of the protein–protein interactions among the non-redundant *E. coli* targets associated with pectin, hesperidin, and eriocitrin. Nodes represent proteins and edges denote high-confidence functional associations. The network reveals densely connected clusters corresponding to major cellular processes, indicating functional interconnectivity among the predicted targets. Subsequently, separate PPI networks were constructed for the 29, 30, and 46 *E. coli* targets identified for eriocitrin, hesperidin, and pectin (Supplementary Figures S1–S3), respectively, to examine the compound-specific interaction landscapes.

The *E. coli*-specific PPI network comprised 134 nodes (84 query proteins + 50 added interactors), each representing a protein (Figure 10). The observed edge count (538) is substantially higher than the expected count (189) for a random set of the same size, and the PPI enrichment p < 10^-16^ indicates that the proteins are significantly more interconnected than expected by chance. This dense connectivity supports the interpretation that the compound-associated proteins participate in shared biological processes and form functionally coherent modules rather than being random, isolated proteins.

### Enrichment analysis of combined (non-redundant) PPI network

GO-BP enrichment of the combined network highlights strong overrepresentation of metabolic and biosynthetic processes (Table 3). The most significant GO-BP terms include: GO:0044281 — Small molecule metabolic process (FDR = 3.07 × 10^−15^), GO:0055086 — Nucleobase-containing small molecule metabolic process (FDR = 5.83 × 10^−14^), GO:0008152 — Metabolic process (FDR = 1.62 × 10^−13^), GO:0044238 — Primary metabolic process (FDR = 5.48 × 10^−11^), and GO:0006099 — Tricarboxylic acid cycle (FDR = 6.36 × 10^−9^). Additional enriched terms include nucleoside/nucleotide metabolic processes, organophosphate metabolism, and cellular respiration-related processes (Table 3).

**TABLE 3:** Enriched GO biological processes in the *E. coli* specific protein-protein interaction (PPI) network.

#Term ID	Term description	False discovery rate
GO:0044281	Small molecule metabolic process	3.07E-15
GO:0055086	Nucleobase-containing small molecule metabolic process	5.83E-14
GO:0008152	Metabolic process	1.62E-13
GO:0044238	Primary metabolic process	5.48E-11
GO:0071704	Organic substance metabolic process	4.90E-09
GO:0044237	Cellular metabolic process	5.82E-09
GO:1901135	Carbohydrate derivative metabolic process	5.82E-09
GO:0006099	Tricarboxylic acid cycle	6.36E-09
GO:0009116	Nucleoside metabolic process	2.22E-08
GO:0006753	Nucleoside phosphate metabolic process	2.54E-08
GO:0009117	Nucleotide metabolic process	6.92E-08
GO:0009123	Nucleoside monophosphate metabolic process	8.13E-08
GO:0009124	Nucleoside monophosphate biosynthetic process	1.12E-07
GO:0009165	Nucleotide biosynthetic process	4.07E-07
GO:0019637	Organophosphate metabolic process	4.07E-07
GO:0042278	Purine nucleoside metabolic process	6.14E-07
GO:0043094	Cellular metabolic compound salvage	6.14E-07
GO:1901137	Carbohydrate derivative biosynthetic process	1.22E-06
GO:0090407	Organophosphate biosynthetic process	1.36E-06
GO:1901564	Organonitrogen compound metabolic process	1.88E-06
GO:0006725	Cellular aromatic compound metabolic process	4.31E-06
GO:1901576	Organic substance biosynthetic process	5.62E-06
GO:0006796	Phosphate-containing compound metabolic process	6.70E-06
GO:0006793	Phosphorus metabolic process	6.84E-06
GO:0072527	Pyrimidine-containing compound metabolic process	6.84E-06
GO:0009119	Ribonucleoside metabolic process	9.02E-06
GO:0019693	Ribose phosphate metabolic process	9.55E-06
GO:0019439	Aromatic compound catabolic process	1.60E-05
GO:0034654	Nucleobase-containing compound biosynthetic process	1.60E-05
GO:0072521	Purine-containing compound metabolic process	1.62E-05
GO:0009161	Ribonucleoside monophosphate metabolic process	1.80E-05
GO:0006139	Nucleobase-containing compound metabolic process	2.34E-05
GO:0044249	Cellular biosynthetic process	2.80E-05
GO:0044283	Small molecule biosynthetic process	2.80E-05
GO:0009129	Pyrimidine nucleoside monophosphate metabolic process	4.21E-05
GO:0043173	Nucleotide salvage	4.21E-05
GO:0046390	Ribose phosphate biosynthetic process	4.21E-05
GO:0006220	Pyrimidine nucleotide metabolic process	4.51E-05
GO:0046128	Purine ribonucleoside metabolic process	4.51E-05
GO:0046700	Heterocycle catabolic process	4.51E-05
GO:1901566	Organonitrogen compound biosynthetic process	5.27E-05
GO:0006633	Fatty acid biosynthetic process	5.39E-05
GO:0009156	Ribonucleoside monophosphate biosynthetic process	5.39E-05
GO:0046483	Heterocycle metabolic process	5.39E-05
GO:1901360	Organic cyclic compound metabolic process	5.39E-05
GO:0009259	Ribonucleotide metabolic process	7.40E-05
GO:0019438	Aromatic compound biosynthetic process	7.74E-05
GO:0006221	Pyrimidine nucleotide biosynthetic process	0.00011
GO:0009112	Nucleobase metabolic process	0.00012
GO:0009260	Ribonucleotide biosynthetic process	0.00012
GO:0019752	Carboxylic acid metabolic process	0.00012
GO:0009056	Catabolic process	0.00014
GO:0009130	Pyrimidine nucleoside monophosphate biosynthetic process	0.00016
GO:1901362	Organic cyclic compound biosynthetic process	0.00019
GO:1901361	Organic cyclic compound catabolic process	0.0002
GO:0018130	Heterocycle biosynthetic process	0.00025
GO:0034641	Cellular nitrogen compound metabolic process	0.00025
GO:0044270	Cellular nitrogen compound catabolic process	0.00025
GO:0009164	Nucleoside catabolic process	0.00026
GO:0046129	Purine ribonucleoside biosynthetic process	0.00026
GO:0072522	Purine-containing compound biosynthetic process	0.00026
GO:0009120	Deoxyribonucleoside metabolic process	0.00029
GO:0006807	Nitrogen compound metabolic process	0.00031
GO:0072330	Monocarboxylic acid biosynthetic process	0.00041
GO:0009987	Cellular process	0.0006
GO:0046394	Carboxylic acid biosynthetic process	0.00068
GO:0072529	Pyrimidine-containing compound catabolic process	0.00071
GO:1901136	Carbohydrate derivative catabolic process	0.00077
GO:0043648	Dicarboxylic acid metabolic process	0.00083
GO:0015980	Energy derivation by oxidation of organic compounds	0.00088
GO:0045333	Cellular respiration	0.00088
GO:0044271	Cellular nitrogen compound biosynthetic process	0.00096
GO:0006166	Purine ribonucleoside salvage	0.0011
GO:0006213	Pyrimidine nucleoside metabolic process	0.0011
GO:0009168	Purine ribonucleoside monophosphate biosynthetic process	0.0011
GO:0009152	Purine ribonucleotide biosynthetic process	0.0019
GO:0046040	IMP metabolic process	0.0019
GO:0034655	Nucleobase-containing compound catabolic process	0.0023
GO:0072523	Purine-containing compound catabolic process	0.0031
GO:0009150	Purine ribonucleotide metabolic process	0.0032
GO:0006177	GMP biosynthetic process	0.0033
GO:0009177	Pyrimidine deoxyribonucleoside monophosphate biosynthetic process	0.0033
GO:0032262	Pyrimidine nucleotide salvage	0.0033
GO:0006206	Pyrimidine nucleobase metabolic process	0.0036
GO:0009221	Pyrimidine deoxyribonucleotide biosynthetic process	0.0048
GO:0044248	Cellular catabolic process	0.0053
GO:0006091	Generation of precursor metabolites and energy	0.0058
GO:1901575	Organic substance catabolic process	0.0061
GO:0009166	Nucleotide catabolic process	0.0062
GO:0006152	Purine nucleoside catabolic process	0.0069
GO:0042454	Ribonucleoside catabolic process	0.0069
GO:1901565	Organonitrogen compound catabolic process	0.0089
GO:0006097	Glyoxylate cycle	0.0097
GO:0006188	IMP biosynthetic process	0.0097
GO:0032263	GMP salvage	0.0142
GO:0046121	Deoxyribonucleoside catabolic process	0.0142
GO:0046125	Pyrimidine deoxyribonucleoside metabolic process	0.0142
GO:0005975	Carbohydrate metabolic process	0.0171
GO:0009262	Deoxyribonucleotide metabolic process	0.0177
GO:0046113	Nucleobase catabolic process	0.0214
GO:0032264	IMP salvage	0.0227
GO:0046133	Pyrimidine ribonucleoside catabolic process	0.0227
GO:0046049	UMP metabolic process	0.0292
GO:0008610	Lipid biosynthetic process	0.0326
GO:0046100	Hypoxanthine metabolic process	0.0342
GO:0110095	Cellular detoxification of aldehyde	0.0477
GO:0032787	Monocarboxylic acid metabolic process	0.0487

These data indicate strong involvement of the target proteins in small-molecule metabolism, nucleotide metabolism (both purine and pyrimidine), and central energy metabolism (TCA, glycolysis-related processes). This supports the hypothesis that the compounds perturb essential core metabolic functions in *E. coli*.

Similarly, KEGG enrichment of the combined network is also dominated by metabolic pathways (Table 4) including eco01100 — Metabolic pathways (FDR = 1.31 × 10^−34^), eco00240 — Pyrimidine metabolism (FDR = 6.77 × 10^−13^), eco01110 — Biosynthesis of secondary metabolites (FDR = 2.12 × 10^−12^), eco00230 — Purine metabolism (FDR = 3.97 × 10^−12^), eco01120 — Microbial metabolism in diverse environments (FDR = 3.74 × 10^−10^), and eco00020 — Citrate cycle (TCA cycle) (FDR = 3.52 × 10^−9^). Other enriched pathways include fatty-acid biosynthesis, pyruvate metabolism, glyoxylate and dicarboxylate metabolism, and carbon metabolism (Table 4). The KEGG results echo that nucleotide biosynthesis/salvage and central carbon/energy metabolism are major functional themes of the target genes, and imply that the three compounds may impair bacterial replication and energy generation by hitting multiple enzymes in those pathways.

**TABLE 4:** Enriched KEGG pathways in the *E. coli* specific protein-protein interaction (PPI) network.

#Term ID	Term description	False discovery rate
eco01100	Metabolic pathways	1.31E-34
eco00240	Pyrimidine metabolism	6.77E-13
eco01110	Biosynthesis of secondary metabolites	2.12E-12
eco00230	Purine metabolism	3.97E-12
eco01120	Microbial metabolism in diverse environments	3.74E-10
eco00020	Citrate cycle (TCA cycle)	3.52E-09
eco01200	Carbon metabolism	2.66E-08
eco00061	Fatty acid biosynthesis	1.56E-06
eco00620	Pyruvate metabolism	1.56E-06
eco00630	Glyoxylate and dicarboxylate metabolism	5.39E-06
eco01212	Fatty acid metabolism	1.93E-05
eco00250	Alanine, aspartate and glutamate metabolism	0.00027
eco00760	Nicotinate and nicotinamide metabolism	0.00027
eco00520	Amino sugar and nucleotide sugar metabolism	0.0096
eco00270	Cysteine and methionine metabolism	0.0103
eco00780	Biotin metabolism	0.0143
eco00541	O-Antigen nucleotide sugar biosynthesis	0.047

### Compound-specific enrichment patterns in *E. coli*

Although the three compounds shared several metabolic themes, each displayed distinct enrichment signatures reflecting differences in their structural classes. These are briefly described as follows:

- Eriocitrin targets were dominated by processes linked to carbohydrate and nucleotide metabolism, including pyrimidine nucleotide biosynthesis and peptidoglycan biosynthetic process. KEGG enrichment supported this trend, with top hits in Pyrimidine metabolism (eco00240, FDR = 4.9 × 10^−15^) and Peptidoglycan biosynthesis (eco00550, FDR = 5.4 × 10^−10^), suggesting interference with nucleotide and cell wall precursor synthesis (Supplementary Table S4).
- Hesperidin, another flavanone glycoside, exhibited enrichment for fatty acid biosynthetic and metabolic processes (GO:0006633, FDR = 2.6 × 10^−10^) alongside nucleotide metabolism, indicating a broader impact on lipid and nucleoside turnover. KEGG results reinforced this dual influence, highlighting Fatty acid biosynthesis (eco00061, FDR = 6.6 × 10^−12^), Purine metabolism (eco00230, FDR = 3.7 × 10^−14^), and Pyrimidine metabolism (eco00240, FDR = 1.3 × 10^−11^) (Supplementary Table S5).
- Pectin targets primarily enriched primary and small-molecule metabolic processes (GO:0044281, FDR = 1.0 × 10^−6^), particularly nucleotide and carbohydrate metabolism. KEGG analysis identified significant terms, including Carbon metabolism (eco01200, FDR = 0.0018), Pyruvate metabolism (eco00620), and Cysteine and methionine metabolism (eco00270), indicating that pectin-targeted proteins cluster in central carbon and amino acid metabolism (Supplementary Table S6).

Together, these compound-specific results demonstrate that all three natural products converge on *E. coli* metabolic homeostasis, though via distinct biochemical entry points.

Although LP-ZnO nanoparticles exhibited the greatest antibacterial activity against *S. epidermidis*, *E. coli* was selected for the computational analyses because it consistently yielded the largest number of predicted targets across all three phytochemicals, thereby providing the most comprehensive dataset for statistically robust network and enrichment analyses. Nevertheless, the network and enrichment analyses identified only candidate bacterial targets and pathways potentially associated with lemon peel-derived phytochemicals, and direct validation of these predictions was beyond the scope of the current study. Therefore, the present computational analyses should be interpreted as exploratory and hypothesis-generating rather than as direct mechanistic evidence of LP-ZnO nanoparticle activity. Future studies should prioritize the common targets identified in this work, namely ThyA, AraC, MglB, MetB, and DeoD, for detailed computational and experimental validation. In addition, extending species-specific analyses to *S. epidermidis* may help establish a closer relationship between computational predictions and the experimentally observed antibacterial susceptibility profile.

## Conclusion

In recent times, bacteria have developed drug resistance, rendering treatment of infections ineffective and limiting treatment options. This work focuses on antibacterial strategies against pathogenic bacteria that can serve as emerging drug targets, especially for treating multidrug-resistant pathogens. We hypothesise that the newly synthesised biofabricated metal nanoparticles possess antibacterial activity and can inhibit biofilm formation, thereby enabling the development of novel antibiotics with multiple drug targets. Furthermore, computational analyses suggested that major lemon peel-derived phytochemicals involved in nanoparticle synthesis may interact with interconnected bacterial protein networks enriched in core metabolic and biosynthetic pathways. The identification of shared and compound-specific targets provides a hypothesis-generating framework for future mechanistic studies and suggests potential phytochemical-associated contributions to the antibacterial activity of LP-ZnO nanoparticles.

## Data Availability

The original contributions presented in the study are included in the article/Supplementary Material, further inquiries can be directed to the corresponding authors.
