# Supplementary material for: A green approach for synthesizing zinc oxide nanoparticles utilizing aqueous extract of lemon peel: antibacterial evaluation and in silico analysis
Source: Front Chem. 2026 Sep 3;14:1880575. doi: 10.3389/fchem.2026.1880575 (PMC13582301; doi:10.3389/fchem.2026.1880575)
Supplement: Supplementary file 1 [file DataSheet1.docx]

**Figure S1.** Protein–protein interaction network of *E. coli* proteins predicted to interact with eriocitrin. The network highlights interconnected modules reflecting functional groupings within the eriocitrin-specific target set.

**Figure S2.** Protein–protein interaction network of *E. coli* proteins predicted to interact with hesperidin. Distinct clusters indicate coordinated functional associations among hesperidin-specific targets.

**Figure S3.** Protein–protein interaction network of *E. coli* proteins predicted to interact with pectin. The network structure reveals modular organization consistent with central metabolic and biosynthetic processes.
